# In vitro activity of four triazole antifungal drugs against clinically common and uncommon yeast species

**DOI:** 10.18502/cmm.5.4.1949

**Published:** 2019

**Authors:** Narges Aslani, Tahereh Shokohi, Mohammad Reza Ataollahi, Saham Ansari, Yousef Gholampour, Ali Khani Jeihooni, Mohammad Hosein Afsarian

**Affiliations:** 1 Infectious and Tropical Diseases Research Center, Tabriz University of Medical Sciences, Tabriz, Iran; 2 Department of Medical Mycology, School of Medicine, Mazandaran University of Medical Sciences, Sari, Iran; 3 Department of Medical Immunology, School of Medicine, Fasa University of Medical Sciences, Fasa, Iran; 4 Department of Parasitology and Mycology, School of Medicine, Shahid Beheshti University of Medical Sciences, Tehran, Iran; 5 Department of Internal Medicine, School of Medicine, Fasa University of Medical Sciences, Fasa, Iran; 6 Department of Public Health, School of Health, Fasa University of Medical Sciences, Fasa, Iran; 7 Department of Medical Mycology and Parasitology, School of Medicine, Fasa University of Medical Sciences, Fasa, Iran

**Keywords:** Antifungal agents, In vitro susceptibility, Yeast species

## Abstract

**Background and Purpose:**

Incidence of fungal infections caused by opportunistic fungal pathogens, such as yeasts and yeast-like species, has undergone an increase in otherwise healthy individuals. These pathogens account for high mortality and show reduced susceptibility to the routine antifungal drugs. Accordingly, antifungal susceptibility testing is an urgent need in the determination of the susceptibility spectrum of antifungals and selection of appropriate antifungal agents for the management of patients with fungal infection.

**Materials and Methods:**

The present study was conducted on 110 yeast strains belonging to 15 species recovered from clinical specimens. Susceptibility of the isolates to four antifungal drugs (i.e., fluconazole, itraconazole, voriconazole, and posaconazole) was tested according to the Clinical and Laboratory Standards Institute guidelines M27-A3 and M27-S4.

**Results:**

Fluconazole exhibited no activity against 4.3% (n=2) of *C. albicans* isolates, whereas the remaining 44 isolates had a minimum inhibitory concentration (MIC) range of 0.125-4 μg/ml. Voriconazole had the lowest geometric mean MIC (0.03 µg/ml) against all isolated yeast species, followed by posaconazole (0.07 µg/ml), itraconazole (0.10 µg/ml), and fluconazole (0.60 µg/ml). Overall, all of the isolates had reduced voriconazole MICs with a MIC range of 0.016-0.5 μg/ml, except for one isolate of *C. albicans* that had a MIC of 1 μg/ml. *Candida haemulonii* as a multidrug-resistant fungus showed a fluconazole MIC of > 64 μg/ml.

**Conclusion:**

The current study provides insight into the antifungal susceptibility profiles of clinically common and uncommon yeast species to four triazole antifungal agents. According to our findings, voriconazole was the most active agent. Awareness about antifungal susceptibility patterns is highly helpful in the selection of appropriate antifungal drugs and identification of the efficiency of the currently used agents.

## Introduction

Over the last few years, the incidence of fungal infections caused by opportunistic fungal pathogens, such as yeasts and yeast-like species, has witnessed a dramatic increase. The most important yeasts isolated from clinical specimens are *Candida* species. These species infect hospitalized patients, especially those admitted to intensive care units or oncology wards. According to the statistics, invasive *Candida* infection is associated with mortality having a range of 40-70% [1-3]. 

While *Candida albicans *continues to be a major cause of candidiasis, however, the evidence is indicative of the emergence of other *Candida* and uncommon yeast species with high mortality and reduced susceptibility to the currently administered antifungal drugs. Some of these species isolated from different clinical sources include *C. parapsilosis*, *Kluyveromyces marxianus* (*C. kefyr*), *Meyerozyma guilliermondii* (*C. guilliermondii)*,* C. intermedia*,* C. lusitaniae*, *C. haemulonii,* *C. auris,* and atypical forms of *Candida albicans* (i.e., *Candida africana*, *Candida dubliniensis*, and *Candida stellatoidea*) [4-7]. 

The routine antifungal agents for candidiasis treatment are still restricted to polyenes, azoles, and the recently developed echinocandins [8, 9]. Toxic effects of amphotericin B as an efficient polyene antifungal agent have limited the application of this medicine for humans [8]. Minimal side effects and high therapeutic index of azole compounds have made them as first-line therapy for the treatment of *Candida* infections (for many years), antifungal prophylaxis, and empirical or pre-emptive treatment [10]. Nevertheless, the number of *Candida* species with variable susceptibilities or acquired resistance to these antifungal agents has been on a growing trend over the past decade [11].

Determination of the antifungal susceptibility patterns of yeast species isolated from clinical sources and the selection of appropriate antifungal agents can be useful for the management of fungal infection. Regarding this, the current investigation was conducted to evaluate the in vitro antifungal susceptibility of a large number of yeast strains isolated from different clinical sources to four triazole antifungal agents, namely ﬂuconazole, voriconazole, itraconazole, and posaconazole, using microdilution broth method.

## Materials and Methods

***Isolates***

This study was conducted on 110 yeast and yeast-like species isolated from the nail (n=46), skin (n=36), bronchoalveolar lavage (n=9), sputum (n=7), mouth (n=5), mucosa (n=3), vagina (n=2), ear discharge (n=1), and urine (n=1) during 8 months [12]. The isolates were obtained from the Reference Culture Collection of Invasive Fungi Research Center in Sari, Iran. They had been previously identified through polymerase chain reaction-restriction fragment length polymorphism (PCR-RFLP), PCR amplification of *hwp1* gene, and sequencing [12].

***Antifungal susceptibility testing***

In vitro antifungal susceptibility tests were assayed for *Candida* species using minimum inhibitory concentrations (MICs). These isolates had been identified as *C. albicans *(n=46), *C. parapsilosis* (n=17), *C. tropicalis* (n=13), *C. guilliermondii *(n=12), *C. glabrata* (n=4), *P. kudriavzevii* (*C. krusei*; n = 4), *C. famata* (n=3), *K. marxianus* (*C. kefyr*; n=2), *C. haemulonii* (n=2), *C. intermedia* (n=1), *C. sorbosivorans* (n=1), *C. stellatoidea* (n=1), *C. africana* (n=1), *Trichosporon jirovecii* (n=2), and *T. asahii* (n=1). The in vitro antifungal susceptibility testing of these species had been based on broth microdilution method following the M27-A3 and M27-S4 guidelines of the Clinical and Laboratory Standards Institute (CLSI) [13, 14]. 

Fluconazole (*Sigma*-*Aldrich*, USA) was dissolved in deionized-distilled water. Furthermore, itraconazole (*Sigma*-*Aldrich*, USA), voriconazole (*Sigma*-*Aldrich*, USA), and posaconazole (*Sigma*-*Aldrich*, USA) were dissolved in dimethyl sulfoxide (Sigma). Fluconazole was prepared at a final concentration of 0.063-64 μg/ml, while a concentration of 0.016-16 µg/ml was considered for itraconazole, voriconazole, and posaconazole. For the purpose of the study, RPMI 1640 medium containing L-glutamine without bicarbonate (Gibco, UK) buffered to pH 7 with 0.165 mol/l 3-N-morpholinepropanesulfonic acid (MOPS, Sigma) was used. Drug-free and yeast-free controls were also included in the study for comparative purposes. 

Plates were stored at -70°C until they were used. Briefly, all isolates were grown on potato dextrose agar (PDA, Difco, Leeuwarden, the Netherlands) plates at 35°C for up to 3 days. Inoculum suspensions were prepared in a sterile saline solution and then adjusted spectrophotometrically at a wavelength of 530 nm and a percent transmission range of 75-77%. The microdilution plates were incubated at 35°C and read visually after 24 h to determine the MIC values of the antifungal agents. The *P. kudriavzevii* (*C. krusei*) ATCC 6258 and *C. parapsilosis* ATCC 22019 were used as quality control strains, and analysis of these strains was performed with every new batch of MIC plates. The MIC endpoints for all antifungals were defined as the lowest drug concentration causing 50% growth inhibition, compared with the growth of a drug-free control.

***Ethical Statement***

The current study was approved by the Ethics Committee of Fasa University of Medical Sciences ethical code: 93210/D,97,247016), Fasa, Iran, and written informed consent was obtained from the patients.

## Results

***Antifungal susceptibility testing***

Table 1 summarizes the results of the MIC range, geometric mean MIC, MIC_50_, and MIC_90_ of four triazole antifungal drugs against a total of 110 clinically *Candida* species and uncommon yeasts obtained from 14 different *Candida* species and two *Trichosporon* species. However, MIC_90_ was not measured when fewer than nine isolates were available. *Candida albicans* complex isolates (*C. stellatoidea* and *C. africana*) showed high susceptibility to the tested antifungal agents. As the results indicated, fluconazole had the widest range and highest MICs against the isolates (0.063-64 µg/ml). 

The MIC ranges in all clinical strains against antifungal drugs were as follows, in increasing order: posaconazole and voriconazole (0.016-1 µg/ml), itraconazole (0.016-4 µg/ml), and fluconazole (0.063-64 µg/ml). Basically, voriconazole, posaconazole, and itraconazole had low MIC_50 _against all tested clinical strains (Table 1). Overall, in terms of GM MICs, voriconazole was found to be the most active agent against all isolates (n=110), followed by posaconazole in comparison with itraconazole and fluconazole. 

Furthermore, most of *P. kudriavzevii* (*C. krusei*) isolates were resistant to fluconazole but not to voriconazole, posaconazole, or itraconazole. In contrast, *C. guilliermondii*, *C. tropicalis*, and *C. parapsilosis* strains were highly susceptible to fluconazole. Remarkably, *C. guilliermondii* was the most susceptible strain to fluconazole, compared to *C. albicans* and other non-*albicans*. In addition, *C. tropicalis *was the most susceptible strain to voriconazole. However, *P. kudriavzevii *(*C. krusei*) had the highest voriconazole MIC value, compared to all tested strains. 

**Table 1:** *In vitro *susceptibility testing of 110 clinical isolates of yeast species to four triazole antifungal agents (minimum inhibitory concentration range, geometric (G) mean, MIC50, and MIC90 values are expressed in µg/ml)

**MICs (μg/ml)**																						
Strains (no.)	**0.016**	**0.031**	**0.063**	**0.125**	**0.25**	**0.5**	**1**	**2 4**		**8**	**16**	**32**		**64**	**Range**	**MIC50/MIC90**			**Mode**	**G mean**	
drugs																						
**All clinical strains (n = 110)**																						
FLZ															0.063-64		0.5/4	0.5			0.60	
ITZ															0.016-4		0.063/1	0.063			0.10	
VRZ															0.016-1		0.031/0.25	0.016			0.03	
PSZ															0.016-1		0.063/0.5	0.125			0.07	
*C. albicans *(n=46)																						
FLZ				6	7	20	5	3	3	1	1				0.125-16		0.5/4	0.5			0.5	
ITZ	6	8	12	14	2	1		2	1						0.016-4		0.063/0.25	0.125			0.08	
VRZ	20	18	3	2		2	1								0.016-1		0.031/0.063	0.016			0.03	
PSZ	8	7	10	16	1	1	3								0.016-1		0.063/0.125	0.125			0.07	
*C. parapsilosis *(n=17)																						
FLZ			2	6	5	1	1	1	1						0.063-4		0.25/4	0.125			0.25	
ITZ	3	3	7	1	1	1	1								0.016-1		0.063/2	0.063			0.06	
VRZ	9	2	3	1	1	1									0.016-0.5		0.016/0.5	0.016			0.03	
PSZ	6	3	4	1	1	1	1								0.016-1		0.031/1	0.016			0.05	
*C. tropicalis *(n=13)																						
FLZ			2	2	4	2	1	1	1						0.063-4		0.25/2	0.25			0.32	
ITZ	2	3	4	2	1	1									0.016-0.5		0.063/0.25	0.063			0.06	
VRZ	7	4	1	1											0.016-0.125		0.016/0.063	0.016			0.02	
PSZ	6	2	1	2	1	1									0.016-0.5		0.031/0.25	0.016			0.04	
*C. guilliermondii *(n=12)																						
FLZ			2	2	3	3	1	1							0.063-2		0.25/1	-			0.28	
ITZ	2	2	3	3	1	1									0.016-0.5		0.063/0.25	-			0.07	
VRZ	5	2	3	1	1										0.016-0.25		0.031/0.125	0.016			0.03	
PSZ	3	3	2	2	1	1									0.016-0.5		0.031/0.25	-			0.05	
*C. glabrata *(n=4)																						
FLZ									1	1	1	1			4-32		-	-			-	
ITZ					1		1	1	1						0.25-4		-	-			-	
VRZ	1		1		1	1									0.016-0.5		-	-			-	
PSZ			1	1		1	1								0.063-1		-	-			-	
*Pichia kudriavzevii (= C. krusei, *n=4)																						
FLZ												1	3		32-64		-	-			-	
ITZ							1	1	2						1-4		-	-			-	
VRZ					2	2									0.25-0.5		-	-			-	
PSZ					1	1	2								0.25-1		-	-			-	
*C. famata *(n=3)																						
FLZ						2	1								0.5-1		-	-			-	
ITZ			1	2											0.063-0.125		-	-			-	
VRZ		2	1												0.031-0.063		-	-			-	
PSZ		1	1	1											0.031-0.125		-	-			-	
*Kluyveromyces marxianus (= C. kefyr, *n=2)																						
FLZ						1	1								0.5-1		-	-			-	
ITZ				1	1										0.125-0.25		-	-			-	
VRZ		1		1											0.031-0.125		-	-			-	
PSZ			1		1										0.063-0.25		-	-			-	
*C. haemulonii* (n=2)																						
FLZ													2		64		-	-			-	
ITZ							2								1		-	-			-	
VRZ			1	1											0.063-0.125		-	-			-	
PSZ					2										0.25		-	-			-	
*Trichosporon jirovecii *(n=2*)*																						
FLZ						1	1								0.5-1		-	-			-	
ITZ				2											0.125		-	-			-	
VRZ		1	1												0.031-0.063		-	-			-	
PSZ			1	1											0.063-0.125		-	-			-	
*C .intermedia *(n=1)																						
FLZ						1									0.5		-	-			-	
ITZ				1											0.125		-	-			-	
VRZ	1														0.016		-	-			-	
PSZ		1													0.031		-	-			-	
*C. sorbosivorans *(n=1*)*																						
FLZ						1									0.5		-	-			-	
ITZ			1												0.063		-	-			-	
VRZ	1														0.016		-	-			-	
PSZ			1												0.063		-	-			-	
*C. stellatoidea *(n=1)																						
FLZ							1								1		-	-			-	
ITZ				1											0.125		-	-			-	
VRZ	1														0.016		-	-			-	
PSZ		1													0.031		-	-			-	
*C. africana *(n=1)																						
FLZ							1								1		-	-			-	
ITZ						1									0.5		-	-			-	
VRZ	1														0.016		-	-			-	
PSZ		1													0.031		-	-			-	
*Trichosporon asahii* (n=1)																						
FLZ							1								1		-	-			-	
ITZ				1											0.125		-	-			-	
VRZ		1													0.031		-	-			-	
PSZ			1												0.063		-	-			-	

VRZ, voriconazole; FLZ, Fluconazole; ITZ, Itraconazole; PSZ, posaconazole

The MIC_90 _values of fluconazole were 4-log_2_-dilution, 3-log_2_-dilution, and 2-log_2_-dilution less active than those of voriconazole, posaconazole, and itraconazole, respectively. The overall frequency of fluconazole resistance in the evaluated data set was 4.3%. Most of the isolates were susceptible to fluconazole. Notably, 6.5% (n=46), 5.9% (n=17), and 7.7% (n=13) of *C. albicans*, *C. parapsilosis*, and *C. tropicalis* isolates were fluconazole-susceptible dose-dependent (SDD), respectively. In this regard, each of four species of *C. glabrata* was fluconazole-SDD with a MIC value of ≤ 32. 

All tested *C. albicans* isolates had low MICs for posaconazole and itraconazole (MIC_50_=0.063 μg/ml). In the current study, *C. albicans, C. parapsilosis, C. glabrata, *and *P. kudriavzevii *(*C. krusei*) had the resistance rates of 6.5% (3/46), 5.9% (1/17), 50% (2/4), and 75% (3/4) to itraconazole, respectively. All isolates of *C. albicans* showed reduced MICs for voriconazole with a MIC range of 0.016-0.5 μg/ml, except for one isolate that was resistant to voriconazole (MIC=1 μg/ml). However, 4.3% (n=46) of *C. albicans* and 11.8% (n=17) of the *C. parapsilosis* isolates were voriconazole-SDD. 

Notably, *C. haemulonii* as a multidrug-resistant fungus showed a fluconazole MIC of > 64 μg/ml. Moreover, *C. albicans* had elevated GM for fluconazole (0.60 μg/ml) in comparison to that for voriconazole (0.03 μg/ml). Overall, voriconazole had a lower MIC_90_ value (0.25 mg/l) than posaconazole (0.5 mg/l), itraconazole (1 mg/l), and fluconazole (4 mg/l). None of the *T. jirovecii *and* T. asahii *isolates were found to be resistant to fluconazole, itraconazole, posaconazole, or voriconazole.

## Discussion

Frequency of fungal infections caused by opportunistic fungal pathogens, particularly the genus of *Candida*, has undergone a dramatic increase [15, 16]. Epidemiologically, most of the isolates withdrawn from various clinical samples are *C. albicans. *Nevertheless, the elevation of non-*albicans* *Candida* and uncommon yeast species with reduced susceptibility to routine antifungals is a serious problem. This issue is much more complicated when affecting patients with immunodeficiency due to the likelihood of yeast invasion to the deeper tissues, resulting in infection dissemination [17, 18]. 

Therefore, the determination of the antifungal resistance patterns of clinical samples is a vital issue facilitating the selection of appropriate antifungal agents for the treatment of fungal infections and surveillance of resistance to antifungal drugs. Azole compounds are the most frequently used clinical antifungal agents for the treatment of candidiasis. However, with the overuse of these agents, the number of drug-resistant fungal isolates is on a growing trend [19]. Regarding this, the current study was focused on the susceptibilities of various *Candida* species to commonly used azole antifungal agents. The results of the current research demonstrated that fluconazole had desirable activities against most of the isolates. 

Nevertheless, *C. albicans* isolates showed a resistance rate of 4.3% against fluconazole in the present study, which is in concordance with the results reported in other studies (e.g., Almeida et al. [5.5%] and Eksi et al. [5.7%]) [20, 21]. Furthermore, Bhattacharjee reported that all of the *C. albicans* strains isolated from blood cultures were susceptible to fluconazole [22]. The results of a recent study carried out by Aslani et al. showed that resistance to fluconazole in the *C. albicans* strains isolated from the oral cavity of cancer patients was higher (15.9%), compared with the rate observed in the current study (4.3%) [6]. In a study performed by Badiee et al. [23], *C. albicans*, *P. kudriavzevii *(*C. krusei*), *C. glabrata, Kluyveromyces marxianus *(*C. kefyr*)*, C. parapsilosis, *and* C. tropicalis *showed the fluconazole resistance rates of 9.3% (16/172), 95.2% (59/62), 95% (38/40), 5% (2/40), 27.7% (5/18), and 33.3% (2/6), respectively. 

In a population-based study conducted by Wisplinghoff et al. on 1,077 *Candida* species isolated from bloodstream, 0.8% (3/478) of *C. albicans*, 100.0% (202/202) of *C. glabrata*, 2.9% (6/211) of *C. parapsilosis,* and 4.9% (6/123) of *C. tropicalis* were non-susceptible to fluconazole [24]. In addition, Castanheira et al. reported the fluconazole resistance rates of 11.9% and 11.6% for *C. glabrata *and *C. tropicalis,* respectively. They also showed that fluconazole inhibited 94.0% and 88.4% of *C. parapsilosis* and *C. tropicalis* isolates, respectively [25]. 

In line with the present results, Bhattacharjee showed that 66.7% (n=6) of the *C. haemulonii* strains were resistant to fluconazole [22]. In the current study, 3 (6.5%) *C. albicans*, 1 (5.9%) *C. parapsilosis,* and 1 (7.7%) *C. tropicalis* isolates were fluconazole-SDD, respectively. Compared with our findings, Eksi et al. detected dose-dependent susceptibility to fluconazole in 11.3% and 5.2% of *C. albicans* and non-*albicans* *Candida*, respectively [21]. It seems that fluconazole remains an effective antifungal agent against yeast species in spite of its widespread application in Iran. 

Most of the *P.* *kudriavzevii* (*C. krusei*) isolates were detected to be resistant to fluconazole. The decreased susceptibility to fluconazole in *P.* *kudriavzevii* (*C. krusei*) isolates was noted in previous studies. Based on the evidence, *P.* *kudriavzevii* (*C. krusei*) is naturally resistant to antifungal drugs, especially fluconazole [21, 26, 27]. In addition, itraconazole resistance rates of *C. glabrata *and *P. kudriavzevii *(*C. krusei*) were reported as 77.8% (14/18) and 33.3% (3/10), 85% (34/40) and 85.5% (53/62), and 50% (7/14) and 30% (6/18), respectively, in other studies [23, 28, 29]. This rate for *C. albicans* was presented as 15.1% (26/172), 5.4% (2/38), 28% (36/117), 12.7% (35/273), and 11.9% (18/167) in other studies [23, 28, 30, 31]. 

In accordance with other investigations, as determined by MIC_90_ values (0.25 mg/L), voriconazole was the most potent agent among the tested azole antifungals [24, 32]. However, in the current study, 2.2% (1/46) of *C. albicans* isolates with a MIC value of 1 mg/L were resistant to voriconazole. On the other hand, the current results are different from those reported by Bhattacharjee who observed a higher voriconazole resistance rate in *C. albicans* and *C. tropicalis* isolates [22]. 

In the present study, resistance to voriconazole was not observed at any of the non-*albicans* *Candida* isolates. Our data are consistent with those of recent studies performed by Badiee et al. [23] and Yenisehirli et al. [33] reporting a similar voriconazole resistance rate for *Candida* species. In a previous study, Wisplinghoff et al. reported that 0.6% (3/478), 5.0% (1/20), 7.6% (2/211), and 9.8% (4/123) of *C. albicans, P.* *kudriavzevii* (*C. krusei*), *C. parapsilosis, *and *C. tropicalis* were non-susceptible to voriconazole, respectively. Furthermore, Wisplinghoff et al. reported that 16.3% of *C. glabrata* isolates had high voriconazole MIC value, which is higher than the rate reported in other recent investigations [24, 32, 34]. 

Castanheira et al. showed that voriconazole inhibited 99.7%, 99.1%, and 88.4% of the *C. albicans*, *C. parapsilosis,* and *C. tropicalis* of the isolates, respectively. In addition, Castanheira et al. reported that voriconazole (MIC_50/90_= 0.25/0.25 μg/mL) was active against all 49 *C. krusei* isolates [25]. In our previous study performed in Iran, the resistance rates of *C. albicans* strains to fluconazole, itraconazole, and voriconazole were obtained as 9.1%, 11.3% and 9.1%, respectively [35]. In another study, we reported fluconazole, itraconazole, and voriconazole resistance rates of 10%, 72.5%, and 37.5% for *C. glabrata* isolates, respectively [36]. 

No breakpoint has been mentioned for posaconazole in the CLSI M27-S4 reference [14]. In this study, the highest posaconazole MIC_90_ value was observed against *C. albicans*, *C. glabrata*,* P. kudriavzevii *(*C. krusei*)*, *and *C. parapsilosis* (1 µg/ml). This was reported as 2 µg/ml in other studies [28, 37, 38]. In a study performed by Wisplinghoff et al., posaconazole MIC_50_ and MIC_90 _values against *C. glabrata* were higher than former reports [24]. Similar to other studies, voriconazole and posaconazole had greater activities against most *Candida* species in comparison to fluconazole [33, 39].

## Conclusion

With the growth of resistant yeast species to routine antifungal agents, the selection of the most appropriate antifungal agent and effective treatment is a critical issue in clinical practice. According to the results of the present study, voriconazole with a low resistance rate might be used as the drug of choice for the treatment of the infections occurring as a result of *Candida* species. Regarding this, it is required to perform further studies in each region to determine the antifungal susceptibility patterns of yeast species for the successful treatment of patients with *Candida* infection.
